# Metformin treatment after the hypoxia-ischemia attenuates brain injury in newborn rats

**DOI:** 10.18632/oncotarget.20779

**Published:** 2017-09-08

**Authors:** Mingchu Fang, Huai Jiang, Lixia Ye, Chenchen Cai, Yingying Hu, Shulin Pan, Peijun Li, Jian Xiao, Zhenlang Lin

**Affiliations:** ^1^ Department of Neonatology, The Second Affiliated Hospital and Yuying Children’s Hospital, Wenzhou Medical University, Wenzhou, Zhejiang 325027, China; ^2^ Center for Neuroscience Research, Children’s National Health System, Washington, DC 20010, United States; ^3^ Molecular Pharmacology Research Center, School of Pharmacy, Wenzhou Medical University, Wenzhou, Zhejiang 325035, China

**Keywords:** metformin, neonatal hypoxic-ischemic brain injury, neuronal apoptosis, neuroinflammation, blood-brain barrier

## Abstract

Neonatal hypoxic-ischemic (HI) brain injury is a devastating disease that often leads to death and detrimental neurological deficits. The present study was designed to evaluate the ability of metformin to provide neuroprotection in a model of neonatal hypoxic-ischemic brain injury and to study the associated molecular mechanisms behind these protective effects. Here, we found that metformin treatment remarkably attenuated brain infarct volumes and brain edema at 24 h after HI injury, and the neuroprotection of metformin was associated with inhibition of neuronal apoptosis, suppression of the neuroinflammation and amelioration of the blood brain barrier breakdown. Additionally, metformin treatment conferred long-term protective against brain damage at 7 d after HI injury. Our study indicates that metformin treatment protects against neonatal hypoxic-ischemic brain injury and thus has potential as a therapy for this disease.

## INTRODUCTION

Neonatal hypoxic-ischemic (HI) brain injury remains a major contributor to high mortality and lifelong morbidity in neonates, along with a reported incidence of 1-8 per 1000 live births in developed countries and 26 per 1000 live births in developing countries [1]. Approximately 10% to 20% of affected infants will die during the postnatal period, and up to 25% of the survivors will develop long-term and irreversible neurological deficits, including neurodevelopmental impairment, epilepsy, learning difficulties and cerebral palsy [2]. These catastrophic neurologic impairments not only significantly affect quality of life for patients but also cause a tremendous economic burden to family and society. To date, the utility of therapeutic hypothermia in the reduction of death and disability is well established as standard treatment for neonatal encephalopathy [3], but this therapy is moderately neuroprotective and merely effective in neonates born at or near term with a short therapeutic window of 6 h [4, 5]. Therefore, better and more effective treatment strategies are urgently needed to prevent or ameliorate neonatal brain damage after HI.

Previous studies have demonstrated that inflammation plays a vital role in the development and exacerbation of the neonatal brain damage induced by hypoxic-ischemic injury [6]. Neonatal hypoxia-ischemia triggers inflammatory processes in the brain, and the initial inflammatory response is thought to depend on activation of innate immune receptors [7]. Toll-like receptors (TLRs) are first-line molecules for initiating innate immune responses. Among all TLRs, Toll-like receptor4 (TLR4) has been shown to widely express on microglia, and mediates neuroinflammatory diseases through activation of innate immunity [8]. Recent studies have shown that the expression of TLR4 is up-regulated in a neonatal rat model of hypoxic-ischemic brain injury [9] and in microglia exposed to hypoxic treatment *in vitro* [10]. Furthermore, activated microglial TLR4 expression can promote nuclear translocation of the nuclear factor-κB (NF-κB) and subsequent production of pro-inflammtory cytokines involved in neurotoxicity [10]. Thus, TLR4/NF-κB signaling pathway provides the potential therapeutic target for neonatal hypoxic-ischemic brain damage.

The blood-brain barrier (BBB) is formed by endothelial cells (ECs) and their accessory structures, including the basement membrane, pericytes, astrocytic end-feet processes. It’s the specialized multicellular structure that determines the barrier function of BBB, which helps to tightly restrict the passage of molecules and cells between blood and brain, and maintain a homeostatic microenvironment that allow ensure proper neuronal function [11]. There is a good amount of evidence that neuroinflammation and oxidative stress are two major contributors to disruption of the vasculature integrity of BBB and the hyperpermeability of BBB after ischemia insult [12, 13]. Furthermore, the pro-inflammatory factors produced within the injury site may cross the disrupted BBB to recruit peripheral immune cells [11], and immune cells infiltration, toxins and detrimental substances penetration may exacerbate the damage of brain parenchyma, including neurons and glia. Therefore, it’s critical to preserve the BBB integrity for the neonatal brain after HI injury.

Substantial data has documented that apoptosis plays a prominent part in occurrence and evolution of central nervous system diseases and injury, notably the neonatal brain damage following HI [14]. In addition, apoptosis is known to be more prominent in the immature than in the juvenile and adult brains [14, 15]. Apoptosis is an critical component involved in cell death following neonatal HI [16], which elicits the delayed cell death in the developing brain that results in a tremendous proportion of cell loss and neurodegeneration [17]. Thus, inhibition of apoptosis in the neonatal brain following HI to reduce the brain damage is of great importance.

Metformin is a biguanide widely prescribed for the therapy of type 2 diabetes mellitus and metabolic syndrome [18]. Previous studies have demonstrated that metformin exhibits a diverse range of pharmacological activities, such as anti-oxidant, anti-inflammatory, anti-apoptosis, anti-tumor properties [19-21]. *In vivo*, metformin has been found to pass through the blood-brain barrier and accumulate in the brain [22]. Recently, metformin has been reported to exert neuroprotective effects in a variety of animal models of central nervous system diseases such as spinal cord injury, and cerebral ischemia/reperfusion injury via regulating inflammatory response, neuronal apoptosis, oxidative stress [23-25]. Furthermore, it has been illustrated that metformin can promote neurogenesis and protect BBB integrity in experimental stroke [26, 27]. Additionally, metformin has been identified to ameliorate cognitive impairments via improve remyelination in a neonatal white matter injury paradigm [28]. However, to the best of our knowledge, few studies have reported that treatment with metformin is able to promote neurogenesis and preserve BBB integrity in an animal model of neonatal HI brain injury. Thus, the neuroprotective effects of metformin and the precise mechanism by which metformin treatment within HI induced brain injury in neonatal rats should been well explored.

In our research, we designed to test whether treatment with metformin immediately after HI injury was able to exert neuroprotective effects on neonatal brain injury in rats. And if neuroprotection of metformin to neonatal HI brain injury was identified, we would further explore whether metformin attenuated neonatal brain damage following HI by inhibiting neuroinflammation, protecting the integrity of the BBB and reducing the neuronal apoptosis.

## RESULTS

### Metformin treatment reduces infarct volume and ameliorates brain edema

**Figure 1:**
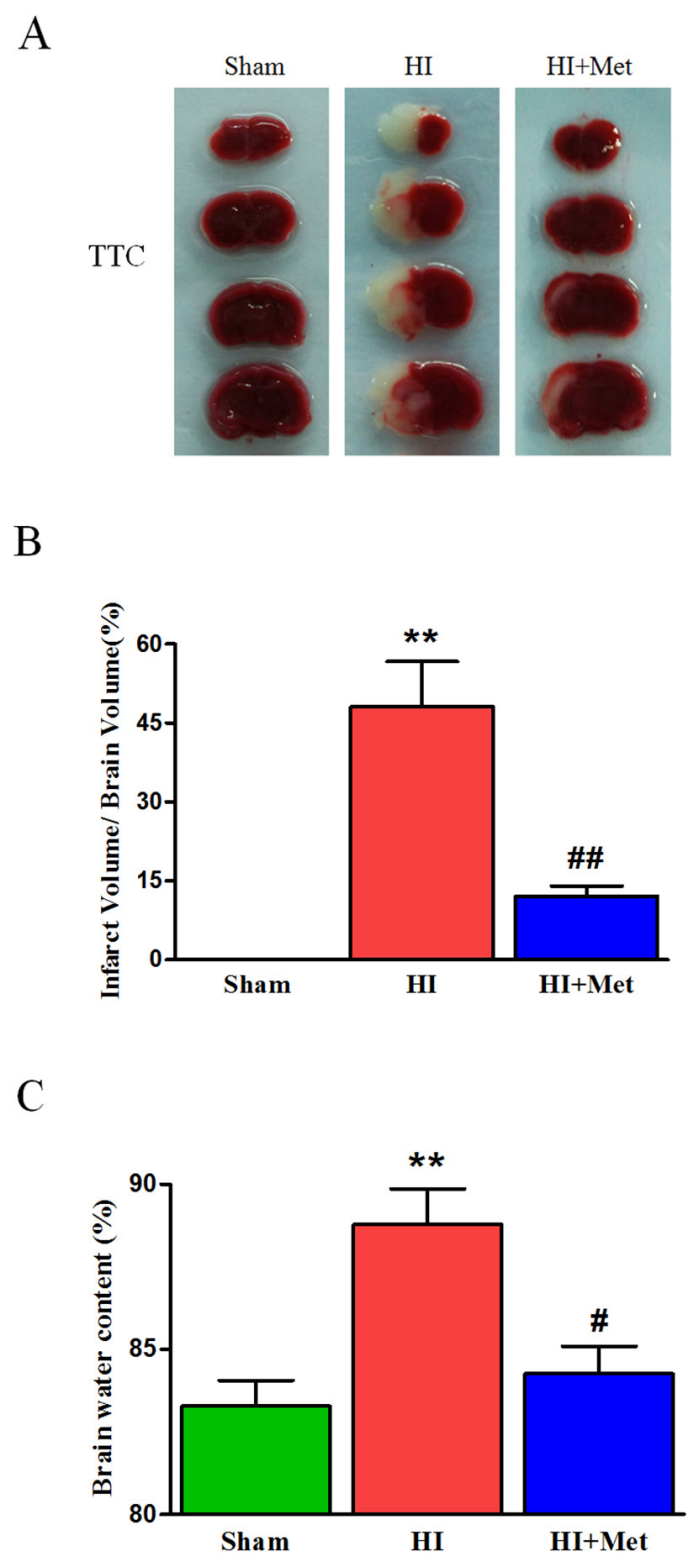
Metformin treatment ameliorated the infarct volume and brain edema in HI brain injury **(A)** Representative TTC stained coronal brain sections from Sham, vehicle-treated HI and metformin-treated HI groups at 24 h after HI were shown. Number of animals, n=5 for each group. **(B)** Quantitative analysis of infarct volume; results were expressed as infarct area/ total area of the ipsilateral hemisphere. **P < 0.01 versus the sham group. ^##^*P* < 0.01 versus the HI group. Values represent the mean±SEM, Number of animals. n=5 for each group. **(C)** Quantification of brain water content in the ipsilateral brain hemisphere at 24 h after HI. **P< 0.01 versus the sham group. ^#^*P* < 0.05 versus the HI group. Values represent the mean±SEM. Number of animals, n=5 for each group.

To make sure whether treatment of metformin was able to exert a protective effect on neonatal rats following hypoxia-ischemia, metformin was administered immediately after HI, the infarct volume was measured to evaluate the extent of brain damage at 24 h post HI via TTC staining. The infarct volume was remarkably reduced from 48.12±14.79% in vehicle-treated HI rat pups to 12.07±3.40% in metformin-treated HI rat pups (Figure 1A and 1B). However, the differences of infarct volume were no significant between sham group and metformin-treatment group. Therefore, the results revealed that treatment of metformin could significantly protect the neonatal brain from hypoxia-ischemia in rats.

To evaluate brain edema, brain water content was detected at 24 h after HI. The results showed that the ipsilateral brain water content of vehicle-treated HI group was significantly increased compared with the sham group (88.79±2.41%vs. 83.28±1.75%, Figure 1C), metformin-treated HI group exhibited an intense reduction in the percentage of brain water content compared with vehicle-treated HI group (84.26±1.86%vs.88.79±2.41%, Figure 1C). The data indicated that metformin treatment obviously attenuated the brain edema after HI injury in neonatal rats.

### Metformin treatment attenuates HI-induced neuronal cell apoptosis and inhibits apoptosis-related protein expression post-HI insult

**Figure 2:**
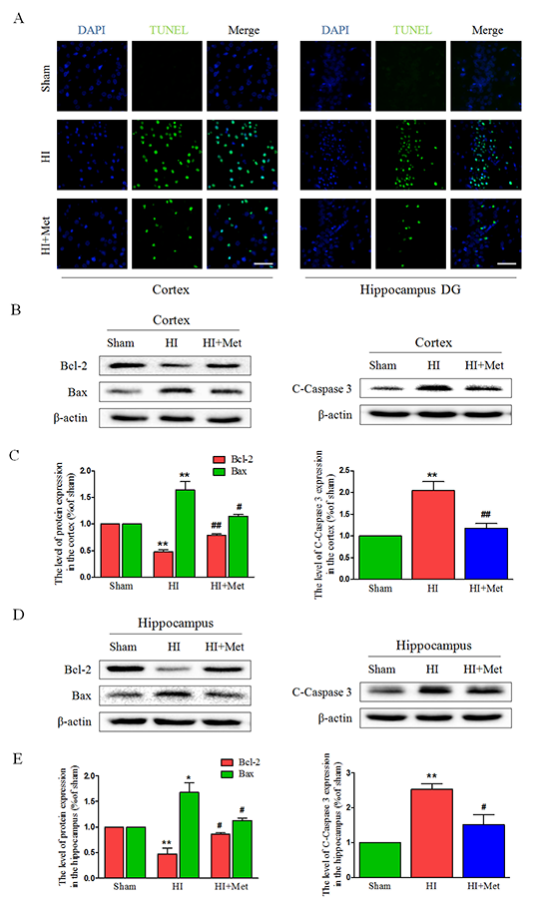
Metformin treatment attenuated apoptosis induced by HI injury **(A)** Representative TUNEL-stained (*green*) and DAPI-stained (*blue*) brain sections in the cortex and hippocampus at 24 h post HI injury. Scale bar = 50 μm. **(B, D)** Representative western blots of apoptosis-related protein, such as Bcl-2, Bax, Cleaved caspase 3 in the cortex (B) and hippocampus (D) at 24 h post HI injury. **(C, E)** Quantification of western blot data from B and D, respectively. *P< 0.05, **P < 0.01 versus the sham group. ^#^*P*< 0.05, ^##^*P*< 0.01versus the HI group. Values represent the mean±SEM, Number of animals. n=5 for each group.

To explore whether metformin could reduce neuronal apoptosis, the apoptosis-related protein expression were assessed by western blot, and the TUNEL staining was performed. Western blot analysis revealed that treatment of metformin obviously reversed the HI-induced increasing level of cleaved caspase 3 in the cortex (Figure 2B and 2C) and hippocampus (Figure 2D and 2E); Similarly, metformin intervention significantly promoted anti-apoptosis protein Bcl-2 expression and inhibited pro-apoptosis protein Bax expression in the cortex (Figure 2B and 2C) and hippocampus (Figure 2D and 2E) compared with vehicle-treated HI group. Furthermore, TUNEL staining was performed to measure the neuronal apoptosis. Both in the hippocampus (Figure 2A) and cortex (Figure 2A), the number of TUNEL-positive cells increased remarkably in vehicle-treated HI group 24 h after HI injury. Comparing to the vehicle-treated HI group, significant reduction of TUNEL-positive cells was detected among the metformin-treated HI group. All these data indicated that the neuroprotective effects of metformin against HI injury was partially through inhibition of neuronal apoptosis.

### Metformin treatment preserves the degradation of tight junction and adherens junction proteins after neonatal HI injury

**Figure 3:**
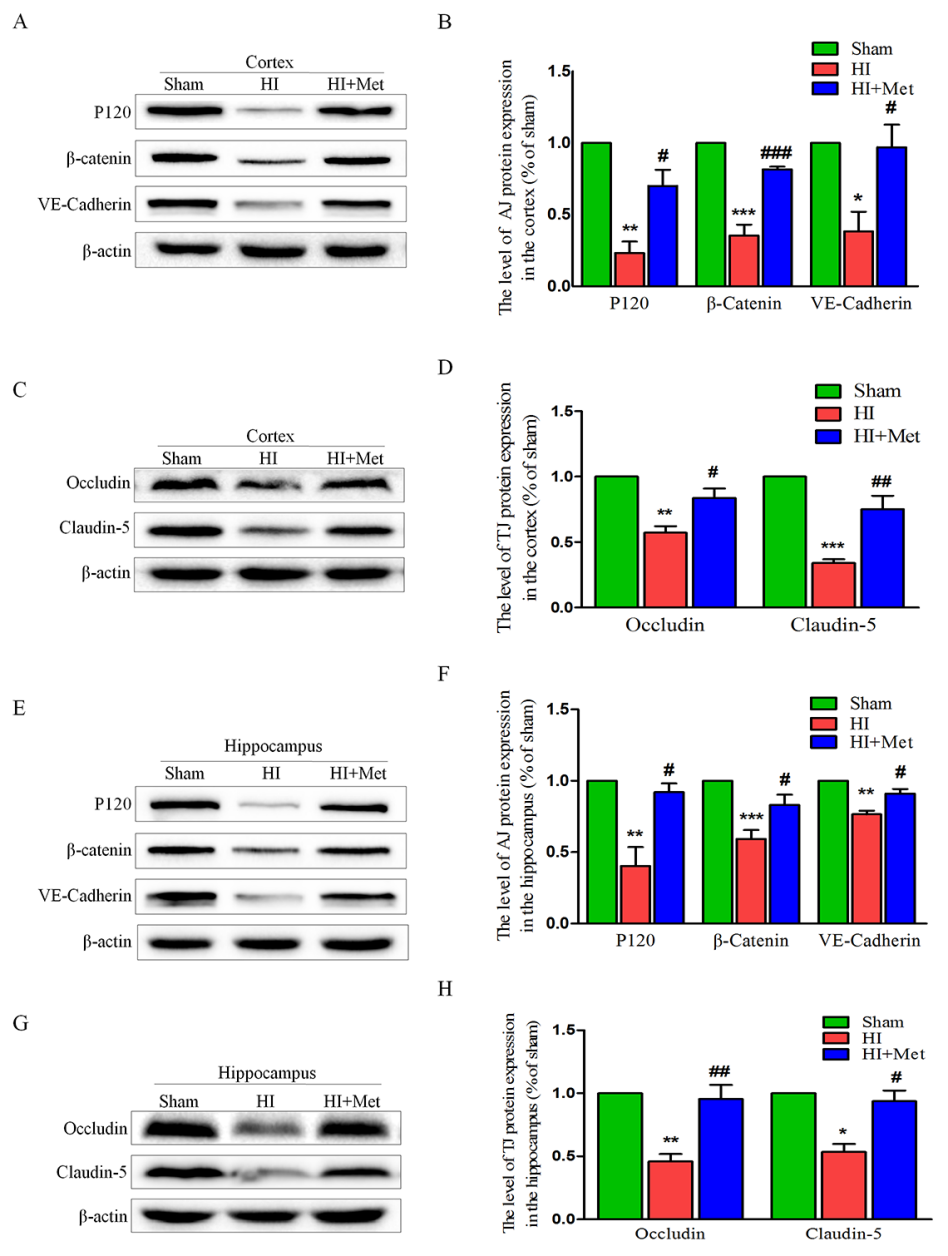
Metformin treatment prevented the degradation of tight junction and adherens junction proteins after HI injury Representative western blots of adherens junction proteins (P120, VE-Cadherin and β-Catenin) in the cortex **(A)** and hippocampus **(E)** at 24 h after HI injury. **(B** and **F)** Quantification of western blot data from A and E. *P< 0.05, **P < 0.01, ***P < 0.001 versus the sham group. ^#^*P*< 0.05, ^###^*P* < 0.001versus the HI group. Values represent the mean±SEM, Number of animals. n=5 for each group. Representative western blots of adherens junction proteins (Occludin and Claudin-5) in the cortex **(C)** and hippocampus **(G)** at 24 h after HI injury. **(D, H)** Quantification of western blot data from C and G. *P< 0.05, **P < 0.01, ***P < 0.001 versus the sham group. ^#^*P*< 0.05, ^##^*P* < 0.01 versus the HI group. Values represent the mean±SEM, Number of animals. n=5 for each group.

The tight junction (TJ) and adherens junction (AJ) are essential for the proper maintenance of BBB integrity. To evaluate whether the metformin prevents the loss of these junctions in neonatal brain following HI, the protein expression of tight junction and adherens junction proteins were measured by western blot analysis. In the vehicle-treated HI group, we observed reduced expression levels of TJ proteins (Occludin and Claudin-5) and AJ proteins (P120-Catenin, VE-Cadherin and β-Catenin) in the cortex (Figure 3A-3D) and hippocampus (Figure 3E-3H), respectively. However, metformin treatment dramatically up-regulated the TJ and AJ proteins to levels similar to those of the sham group (Figure 3). These data showed that metformin preserved the BBB integrity in neonatal rats following HI, which was associated with the significantly decreased degradation of TJ and AJ proteins.

### Metformin treatment prevents the loss of pericytes after neonatal HI injury

**Figure 4:**
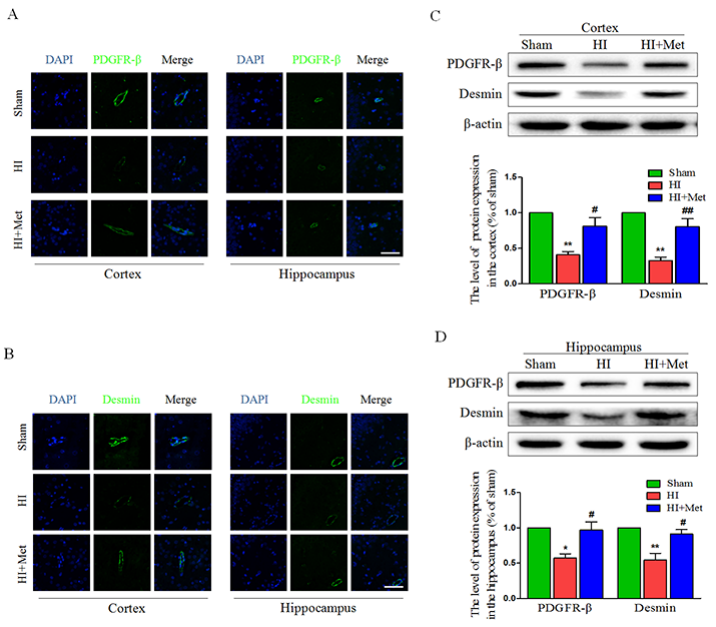
Metformin treatment prevented the loss of pericytes after HI injury **(A)** Representative micrographs showing immunofluorescence staining of PDGFR-β (green) in the hippocampus and cortex, and nucleus (blue) was labeled with DAPI. Scale bar = 50 μm. **(B)** Representative micrographs showing immunofluorescence staining of PDGFR-β (green) in the hippocampus and cortex, and nucleus (blue) was labeled with DAPI. Scale bar = 50 μm. **(C, D)** Representative western blots and quantification data of PDGFR-β and Desmin in the cortex (C) and hippocampus (D) at 24 h after HI injury. *P< 0.05, **P < 0.01 versus the sham group. ^#^*P*< 0.05, ^##^*P* < 0.01 versus the HI group. Values represent the mean±SEM, Number of animals. n=5 for each group.

Pericytes are vascular mural cells embedded in the basement membrane of blood microvessels [29]. In the CNS, pericytes exhibit diverse functional activities, including maintaining the integrity of BBB, promoting angiogenesis, controlling endothelial cell-mediated leukocyte adhesion and transmigration into the CNS [30]. To investigate the role of metformin in the loss of pericytes in the neonatal rats brain following HI, we tested the abundance of pericytes in the brain by detecting the pericyte markers, including PDGFR-β and desmin. Immunofluorescence staining of PDGFR-β (Figure 4A) and desmin (Figure 4B) in the cortex and hippocampus showed decreased numbers of pericytes in HI-induced neonatal rats, which was remarkably reversed by treatment with metformin. The PDGFR-β and desmin western blot analysis results corresponded with the Immunofluorescence staining of the same marker. The western blot analysis showed that the protein levels of PDGFR-β and desmin both in the cortex (Figure 4C) and hippocampus (Figure 4D) were significantly decreased in the vehicle-treated HI group when compared with the sham group, but were extensively up-regulated by treatment with metformin. These results demonstrated that metformin preserved the BBB was partially due to improved pericytes survival in neonatal rats after HI injury.

### Metformin treatment reduces the activation of astrocyte and microglia after neonatal HI injury

**Figure 5:**
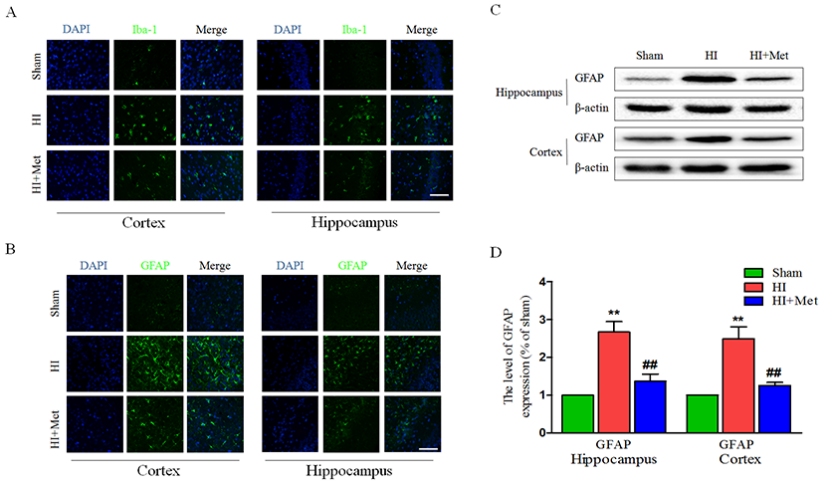
Metformin treatment reduced astrogliosis and microgliosis after HI injury **(A)** Representative micrographs showing immunofluorescence staining of Iba-1 (green) in hippocampus and cortex, and nucleus (blue) was labeled with DAPI. Scale bar = 50 μm. **(B)** Representative micrographs showing immunofluorescence staining of GFAP (green) in the hippocampus and cortex, and nucleus (blue) was labeled with DAPI. Scale bar = 50 μm. **(C)** Representative western blots of GFAP in cortex and hippocampus at 24 h after HI injury. **(D)** Quantification of western blot data from C. **P < 0.01 versus the sham group. ^##^*P* < 0.01 versus the HI group. Values represent the mean±SEM, Number of animals. n=5 for each group.

Previous studies have acknowledged that astrocytes and microglia were activated and accumulated in the lesion site when the neonatal rats received a HI insult, astrogliosis and microgliosis would induce and aggravate the brain injury post HI. To verify whether metformin attenuates the activation of astrocyte and microglia in the cortex and hippocampus, the levels of GFAP (a marker of astrocyte) and Iba-1 (a marker of microglia) were detected by immunofluorescence staining and the protein expression of GFAP was measured by western blot analysis. Compared with the sham group, the number of Iba-1 positive microglia (Figure 5A) and GFAP positive astrocyte (Figure 5B) was notably elevated in the cortex and hippocampus, respectively. In contrast, the number of Iba-1 positive microglia and GFAP positive astrocyte was significantly decreased in the metformin-treated HI group (Figure 5A and 5B), indicating that the activation of microglia and astrocyte induced by HI was ameliorated by treatment of metformin. The results of GFAP immunostaining was further confirmed by western blot analysis of the same marker. The enormously elevated protein level of GFAP (Figure 5C and 5D) resulted from HI insult was down-regulated by treatment of metformin.

### Metformin treatment down-regulates the neuroinflammation by inhibiting the TLR4/ NF-κB signaling pathway after neonatal HI injury

**Figure 6:**
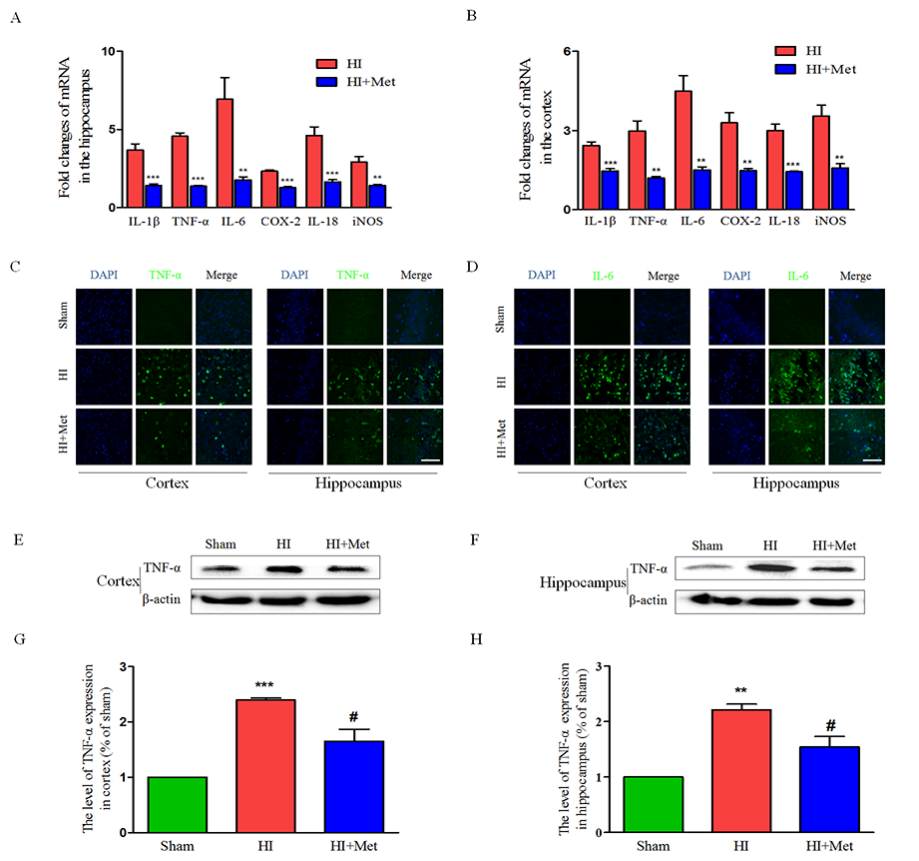
Metformin treatment decreased the expression of inflammatory factors at both the mRNA and protein levels The levels of mRNA expression in cortex **(B)** and hippocampus **(A)** at 24 h after HI injury are reported as the value normalized to β-actin for each sample. **P < 0.01, ***P < 0.001 versus HI group. Values represent the mean±SEM, Number of animals. n=5 for each group. **(C)** Representative micrographs showing immunofluorescence staining of TNF-α (green) in the hippocampus and cortex, and nucleus (blue) was labeled with DAPI. Scale bar = 50 μm. **(D)** Representative micrographs showing immunofluorescence staining of IL-6 (green) in the hippocampus and cortex, and nucleus (blue) was labeled with DAPI. Scale bar = 50 μm. Representative western blots and quantification data of TNF-α in cortex **(E** and **G)** and hippocampus **(F** and **H)** at 24 h after HI injury. **P < 0.01, ***P < 0.001 versus the sham group. ^#^*P* < 0.05 versus the HI group. Values represent the mean±SEM, Number of animals. n=5 for each group.

It has been reported that metformin inhibited the neuroinflammation in the animal models of central nervous system diseases [20, 21, 27]. To evaluate the effects of metformin on HI-induced inflammation in neonatal rats, several inflammatory markers were detected at 24 h after HI injury, at which inflammatory response can be reliably measured but the brain damage has not yet happened [31]. First, at 24 h after HI, the mRNA expression levels of TNF-α, IL-1β, IL-6, IL-18, COX-2 and iNOS in the cortex and hippocampus were measured by real-time RT-PCR. Real-time RT-PCR analysis indicated that the HI group treated with saline exhibited up-regulated expression of TNF-α, IL-1β, IL-6, IL-18, COX-2 and iNOS in the cortex (Figure 6B) and hippocampus (Figure 6A) compared with the sham group. However, mRNA levels of these inflammatory factors remarkably decreased in the metformin-treated HI group (Figure 6A and 6B). Western blot analysis and immunofluorescence staining were then used to examine the expression of the inflammatory cytokines in the cortex and hippocampus. Immunofluorescence staining assay indicated that there were slight expression of IL-6 (Figure 6D) and TNF-α (Figure 6C) in the cortex and hippocampus in the sham group, whereas strong positive immunoreactivity of IL-6 and TNF-α were found in the cortex and hippocampus after HI (Figure 6C and 6D). In the metformin-treated HI group (Figure 6C and 6D), the fluorescence signal of IL-6 and TNF-α were respectively decreased. Furthermore, the result of TNF-α western blot analysis was consistent with the immunofluorescence of the same marker. The protein expression of TNF-α in the cortex (Figure 6E and 6G) and hippocampus (Figure 6F and 6H) was expressed at the lowest level in the sham group, and significantly up-regulated after HI. Metformin treatment could mitigate HI-induced increasing expression of TNF-α in the cortex and hippocampus, respectively (Figure 6E-6H). All of these results suggested that inflammatory response was activated by HI, but was suppressed by metformin.

**Figure 7:**
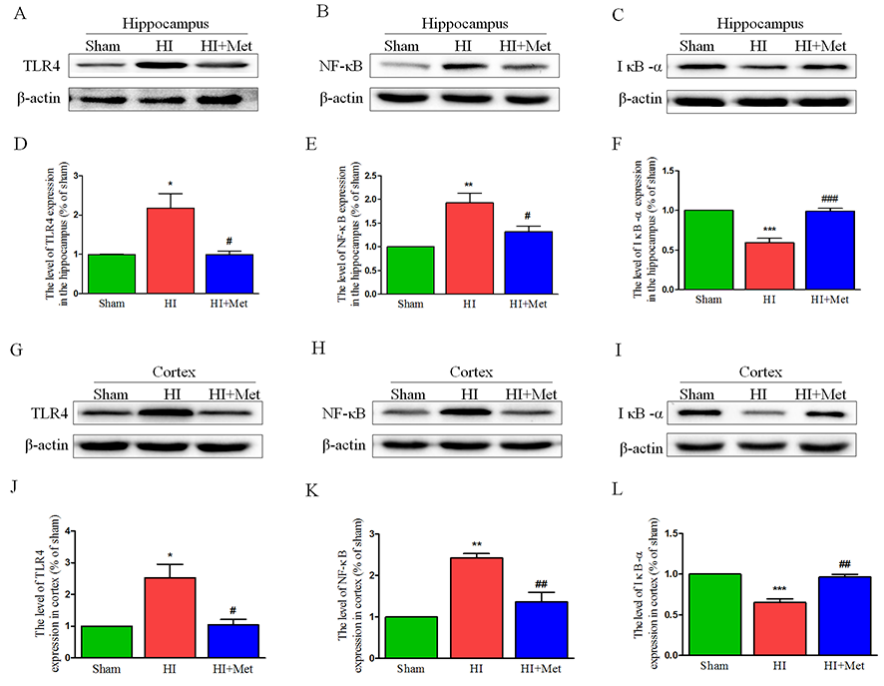
Metformin treatment inhibited TLR4/ NF-κB signaling pathway proteins expression after HI injury Representative western blots of TLR4, NF-κB and IκB-α in the hippocampus **(A-C)** and cortex **(G-I)** at 24 h after HI injury. Quantification of western blot data of TLR4, NF-κB and IκB-α in the hippocampus **(D-F)** and cortex **(J-L)**. *P < 0.05, **P < 0.01, ***P < 0.001 versus the sham group. ^#^*P* < 0.05, ^##^*P* < 0.01, ^###^*P* < 0.001 versus the HI group. Values represent the mean±SEM, Number of animals. n=5 for each group.

The results above have shown that metformin exerted an anti-inflammatory effect on neonatal rat brain after HI, but the underlying mechanism by which metformin regulates the neuroinflammation in the setting of neonatal HI brain injury remains obscure. Previous studies have demonstrated that the TLR4/NF-κB signaling pathway plays a vital role in attenuating neuroinflammation in the animal models of CNS diseases. Thus, we tested whether metformin inhibited the neuroinflammation via suppressing TLR4/NF-κB signaling pathway in neonatal rats following HI. Western blot analysis showed that the protein expression of TLR4 and NF-κB in the cortex (Figure 7G-7H and 7J-7K) and hippocampus (Figure 7A-7B and 7D-7E) were extensively increased in the vehicle-treated HI group when compared with the sham group and it was markedly lower in the metformin-treated HI group than the vehicle-treated HI group at 24h after HI injury. In contrast, the protein expression of IκB-α both in the cortex (Figure 7I and 7L) and hippocampus (Figure 7C and 7F) were dramatically down-regulated in vehicle-treated HI group but was significantly up-regulated in metformin-treated HI group when compared with vehicle-treated HI group at 24h after HI injury. These findings suggested that metformin attenuated the neuroinflammation via suppressing TLR4/NF-κB signaling pathway in neonatal rats following HI.

### Long-term neuroprotective effects of metformin treatment

As shown above, metformin treatment exerted short-term (24 h) protective effects on neonatal HI brain injury. To further test the long-term neuroprotection of metformin treatment against the HI-induced brain injury, brain atrophy and ipsilateral brain tissue loss were studied at 7 d post HI insult.

**Figure 8:**
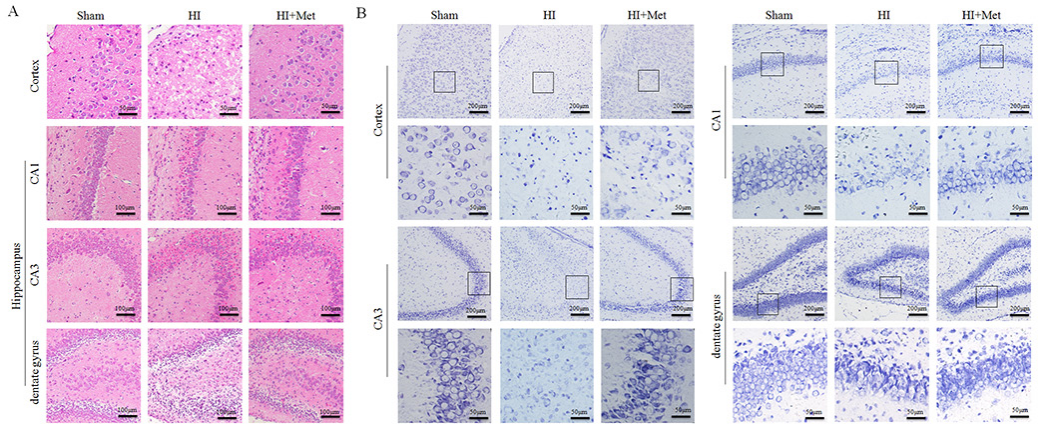
Metformin treatment decreased the damage of tissue structure and the loss of neurons after HI injury **(A)** Representative images of hematoxylin and esosin (H&E) staining in the cortex and hippocampus (CA1, CA2 and dentate gyrus) at 7 d post HI insult. Scale bar = 50 μm, 100μm. **(B)** Representative images of Nissl staining in the cortex and hippocampus (CA1, CA2 and dentate gyrus) at 7 d post HI insult. Scale bar = 50 μm, 200μm.

**Figure 9:**
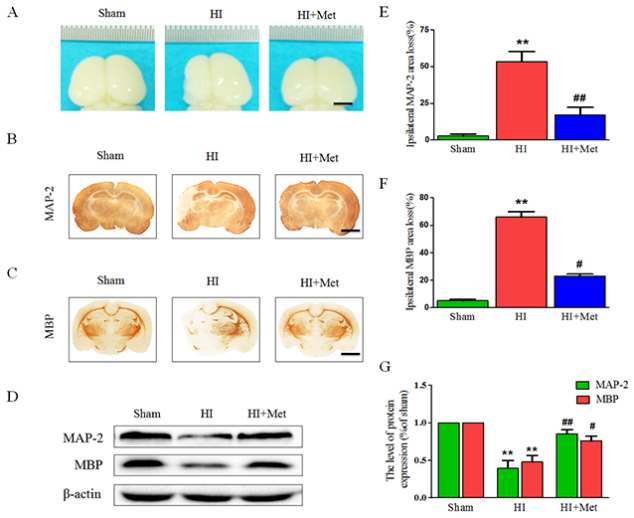
Metformin treatment attenuated brain atrophy, enhanced axonal repairation and promoted remyelination after HI injury Rats were treated with metformin daily for 7 consecutive days, and the brains from each group were acquired at 7 d post HI injury. **(A)** General observation of rat brain from each group at 7 d after HI injury. Scale bar = 1 cm. **(B-C)** Representative images of immunohistochemical staining for MAP-2 (B) and MBP (C). Scale bar = 200 μm. **(D)** Representative western blots of MAP-2 and MBP. **(E-F)** Quantification of ipsilateral MAP-2 and MBP area loss. **P < 0.01 versus the sham group. ^#^*P* < 0.05, ^##^*P* < 0.01 versus the HI group. Values represent the mean±SEM, Number of animals. n=5 for each group. **(G)** Quantification of western blot data from D. **P < 0.01 versus the sham group. ^#^*P* < 0.05, ^##^*P* < 0.01 versus the HI group. Values represent the mean±SEM, Number of animals. n=5 for each group.

First, general morphology of the brain at 7 d post HI insult were observed. As shown in Figure 9A, the ipsilateral hemispheres from the vehicle-treated HI group exhibited atrophy and liquefaction with a distinct collapse, whereas the brain atrophy was ameliorated in the metformin-treated HI group. HE staining and Nissl staining were then performed to assess the histopathological and neuronal alteration in the ipsilateral cortex and hippocampus at 7 d post HI insult. The results were illustrated in Figure 8. In the sham group, neurons in the hippocampal CA1, CA3, dentate gyrus and cortex were oval or round in shape with intact and clear nuclei, and remained well-arranged. In contrast, in the vehicle-treated HI group, shrunken and deformed neurons with pyknotic nulei, disordered neuronal arrangement, decreased neuronal density and even absence of neurons with disappearance of Nissl’s body, were observed in the CA1, CA3, dentate gyrus and cortex. After metformin treatment, the extent of degeneration and necrosis of neuron in the hippocampal CA1, CA3, dentate gyrus and cortex was markedly decreased compared with the vehicle-treated HI group, along with up-regulated neuronal density. In addition, in the metformin-treated HI group, we also found that surviving neurons in the CA1, CA3, dentate gyrus and cortex appeared a well preserved architecture, which was similar to that observed in the sham group.

To gain further insight into long-term protective effects of metformin treatment, we tested whether metformin was capable to promote remyelination and axonal repairation in neonatal rats following HI, the western blot analysis and the immunohistochemistry of MAP-2 (a biomarker for neuron) and MBP (a biomarker for oligodendrocyte) were performed at 7 d post HI insult. Figure 9B, 9C, 9E and 9F) showed the results of immunohistochemical staining revealing that MAP-2-positive and MBP-positive area were extensively decreased in the hemispheres ipsilateral to the ligated side in the vehicle-treated HI group when compared with the sham group, whereas administration of metformin once a day for 7 consecutive days significantly reduced MAP-2-positive and MBP-positive area loss, respectively. In addition, these immunohistochemical staining results were confirmed by the western blot analysis (Figure 9D and 9G). Dramatic reduction of protein expression of MAP-2 and MBP was observed in the vehicle-treated HI group, but was prevented by the metformin treatment. Collectively, these data showed that metformin treatment promoted remyelination and axonal reparation in the neonatal rats following HI.

## DISCUSSION

Hypoxia-ischemia insult to the immature brain will contribute to unrepairable brain damage intimately associated with mortality and severe long-term neurological impairments in newborns. Despite the recent widespread use of hypothermia therapy, approximately half of the cooled infants die or suffer neurodevelopmental disability [32]. Thus, new effective therapeutic approaches are strongly needed. The pathological events of neonatal HI brain injury appear to be heterogeneous, including inflammation, oxidative stress, excitotoxicity, apoptosis, neurovascular dysfunction [6, 15, 33]. Interestingly, the major target of HI injury in the immature brain is the neurovascular unit (NVU), which is comprised of neurons, glial cells and microvessels. Accumulating evidence has acknowledged that neuronal apoptosis, reactive gliosis, and disruption of BBB are closely linked to the neonatal brain damage following lethal HI insult [16, 33].

Here, in this study, we found that metformin treatment remarkably reduced brain damage, inhibited the activation of resident microglia and astrocyte, down-regulated the production of pro-inflammatory mediators, decreased the neuronal apoptosis, and protected the BBB integrity after HI injury in neonatal rats. Our results clearly demonstrated that metformin treatment was effective at amelioration of the neonatal brain injury in rats following HI.

In present study, we demonstrated for the first time that a concentration at 20 mg/kg for a single subcutaneous injection of metformin significantly reduced brain infarct volume and ameliorated the brain edema in newborn rats at 24 h after HI injury, which provided the evidence for a direct neuroprotective role of metformin. Further, we tested whether metformin conferred long-term neuroprotection against neonatal HI brain injury. Our general observations in this study showed that metformin reduced brain tissue loss and attenuated the brain atrophy in the neonatal brain after HI injury. In addition to the general morphology of brain, our data demonstrated that metformin was able to promote neuronal and oligodendrocyte regeneration by administration of metformin once daily for 7 consecutive days, which indicated that metformin exerted the long-term protective effects on not only the gray matter but also the white matter in immature brain. The brain protection from hypoxia-ischemia was achieved at 20 mg/kg, which is in accordance with efficacious single dose (20 mg/kg) in a model of childhood brain injury [34]. Taken together, the neuroprotective role of metformin in the developing brain following HI had been identified in present study.

Inflammation is increasingly recognized as a pivotal role in the neonatal brain damage following HI. Previously published reports have shown that the HI insult immediately elicits the inflammatory response characterized by increased expression of inflammatory factors at both the RNA and protein levels [35]. Tremendous expression of these inflammatory factors in the cortex and hippocampus after HI injury were observed in our experiments, which was conformed to previous studies [36]. In our study, metformin was identified to suppress inflammatory process in neonatal brain after HI by significantly decreasing the mRNA and protein levels of inflammatory factors. These results are in line with the recent findings indicating that metformin exerts anti-inflammatory effects on cerebral inflammation [27].

Pro-inflammatory cytokines in brain injury in neonates following HI are initially derived from the glial cells, especially the activated microglia and astrocytes [6, 36]. In the CNS, Microglia are a major glial component and exhibit the function of immune-surveillance in a resting state. However, when the HI event occurs, microglia are strongly activated and release inflammatory factors contributing to inflammatory process in the brain gray matter [35, 37]. In our study, we did find that the number of Iba-1 immunostaining cells was significantly increased in the cortex and hippocampus after HI, which indicated that microglia were activated by the HI insult. Another subtype of glial cells in the CNS, astrocytes in the brain gray matter response to the HI insult through a process referred to as reactive astrogliosis [37, 38], which was consistent with our experimental observation that the number of GFAP immunostaining cells was increased in the cortex and hippocampus after HI. In addition to the activated microglia, the activated astrocytes also produce pro-inflammatory factors, and rapid up-regulation of these factors exacerbates the brain damage after HI. However, we found that treatment with metformin led to the reduction in the number of Iba-1 and GFAP immunostaining cells in the cortex and hippocampus. These results implied that metformin was able to ameliorate the activation of microglia and astrocyte induced by HI, which was suggested to be responsible for the decreased secretion of pro-inflammatory factors after metformin.

These findings above unambiguously demonstrated that the metformin exhibited anti-neuroinflammation functional property in the neonatal brain injury in rats following HI. In current study, we sought to uncover the underlying molecular mechanisms responsible for the anti-inflammatory effects of metformin in the neonatal brain damage after HI, and clearly demonstrated that and clearly demonstrated that the anti-inflammatory effect of metformin was mediated by suppressing the TLR4/NF-κB signaling pathway.

There are abundant studies showing that TLR4-mediated NF-κB signaling pathway plays a critical role in the initiation of cerebral inflammation in CNS diseases [39, 40]. TLR4 is an innate and adaptive immune cell receptor, which is well known as a mediator of inflammatory reaction involved in neonatal hypoxia brain injury [10]. TLR4 up-regulation has been found in the animal models of neonatal brain injury after HI and in primary microglia exposed to hypoxic treatment *in vitro* [9, 10]. Mice deficient in TLR4 have reduced brain damage and improved neurological and behavioral outcomes after ischemia/reperfusion [41, 42]. NF-κB is a cardinal nuclear transcription factor that regulates the expression of inflammation-related genes. Under normal physiological condition, NF-κB is complexed with its inhibitor of nuclear factor-κB (IκB) in an inactive state and localized in the cytoplasm. when an ischemic event occurs, IκB is phosphorylated and degraded, which induces the activation of NF-κB contributing to transcription of many pro-inflammatory genes that encode cytokines, chemokines, and enzymes such as TNF-α, IL-6, IL-1β and iNOS, these mediators are implicated in the progression of neonatal cerebral inflammation after HI injury [43]. It has been shown in previous animal studies that HI insult induces NF-κB up-regulation in neonatal brain injury [43], whereas inhibition of NF-κB ameliorates the brain damage and improves long-term motor and cognitive outcomes [44]. In addition, it has been well demonstrated that the activation of the TLR4-mediated signaling pathway facilitates activation of the NF-κB pathway, resulting in the production of pro-inflammatory factors and occurence of cerebral inflammation [45]. In the present study, We found that neonatal HI brain injury was associated with a significant increase of TLR4 and NF-κB expression, IκB degradation, and all these results were in accordance with the previous studies [9, 43]. The TLR4 and NF-κB expression, IκB degradation were observed to decrease following metformin administration, which provides strong evidence such that the anti-inflammatory effect of metformin will cause the inhibition of the TLR4/NF-κB signaling pathway in neonatal HI brain injury.

An intact BBB is vital for building and maintaining a microenvironment that allows for proper neuronal function, as well as protects the CNS from injury and disease [46]. The barrier function of BBB is mainly attributable to the brain capillary ECs which form a tight seal attributing to the presence of well-developed apical TJs that control the passage of material between brain and blood [11]. AJs are found deeper in the ECs and function in connecting the actin cytoskeletons of adjacent cells [47]. Thus, both the TJ and AJ play a significant role in the regulation of BBB permeability. Accumulating evidence shows the disruption of the BBB is a devastating event in the pathogenesis of various neurological disorders, including neonatal HI brain injury [48]. Previous studies have demonstrated that neonatal brain damage after HI injury is accompanied by the BBB breakdown [13, 49], characteristic of increased BBB permeability, reduced TJ and AJ proteins and impaired transporter function [49, 50]. Severe brain edema was found in the neonatal rats following HI injury in our experiments, which resulted from the increased permeability after disruption of BBB integrity. In agreement with previous evidence, our current results showed that the disruption of BBB was associated with the degradation of TJ and AJ proteins after HI injury. Interestingly, metformin was able to attenuate the brain edema and reduce the degradation of TJ and AJ proteins, which indicated that metformin prevented HI induced the BBB breakdown partly through decreasing the loss of TJ and AJ proteins. Pericytes are embedded in the basement membrane and attach at irregular intervals along capillary walls. Recent studies have uncovered a critical role for pericytes in the maturation and maintenance of the BBB [51, 52]. Pericytes are required for proper formation of the BBB during embryogenesis and absolute pericyte coverage determine relative vascular permeability [51]. Additionally, it has been found that a set of adult viable pericyte-deficient mouse mutants exhibit up-regulated permeability of the BBB [52]. Furthermore, pericytes regulate functional properties of the BBB, including the formation of tight junction and vesicle trafficking in ECs [51]. Our current findings showed that the protein expression of PDGFRβ and Desmin were reduced in the neonatal brain after HI injury, whereas metformin treatment remarkably increased the protein expression of PDGFRβ and Desmin. These findings implied that metformin protected the BBB integrity against HI was partially associated with increased pericyte survival in neonatal brain after HI injury. Taken together, ECs and pericytes are the therapeutic target for BBB disruption induced by HI in the neonatal brain damage, which are protected against HI insult by metformin in our study.

Apoptosis is one of the forms of programmed cell death, which plays a prominent role in the evolution of hypoxic-ischemic brain injury in neonates [53]. Inhibition of apoptosis has been repeatedly proposed as therapeutic target for neuronal rescue in newborn rats following HI [17]. Recent studies report that metformin mitigates apoptosis and promotes function recovery in several CNS diseases, such as spinal cord injury [25] and cerebral ischemia/reperfusion injury [24]. In present study, we found that metformin provided neuroprotection against HI in neonatal brain partly due to its anti-apoptotic activity. Bcl-2 family plays a significant role in regulating the neuronal apoptosis. Among the members of Bcl-2 family, Bcl-2 is a vital inhibitor of apoptosis that suppresses caspase activity and enhances cell survival, while Bax is a key pro-apoptotic protein that participates in the promotion of apoptosis and leads to cell death [14, 54]. Caspase-3 is one of the most widely studied caspases, and caspase-3 activation following HI event is implicated in the induction of neuronal apoptosis [55]. Our findings that showed down-regulation of Bcl-2 expression and up-regulation of both Bax and cleaved caspase-3 expression after HI in neonatal animals were inhibited by metformin intervention, which indicated that metformin suppressed the initiation of apoptosis to some extent. Furthermore, the DNA fragmentation was measured by TUNEL staining, and the results showed that reduction in the number of TUNEL-positive cells was observed in the neonatal HI brain after metformin treatment, which provided evidence for a direct anti-apoptotic effect of metformin in the neonatal HI brain damage. The anti-apoptotic effect of metformin may partially account for the recovery of brain tissue and neuronal function.

Our study identified that metformin conferred significant neuroprotection against neonatal HI brain injury and clarified that the mechanism by which metformin ameliorated the brain injury after HI. However, there were several limitations to our study. Firstly, we explicitly confirmed that metformin treatment promoted cellular recovery and diminished ultrastructural abnormalities, whereas the effects of metformin treatment on the long-term functional recovery following HI in the neonatal rats were not yet proved. Thus, the behavioral tests such as Morris Water Maze test, complex running wheel task and inclined beam-walking task, all of which will be performed in the future investigations to evaluate functional outcomes. Secondly, metformin exerts neuroprotective effects partially through regulating oxidative stress which is involved in the development of neonatal HI brain injury, but we did not explored it in present study. Additionally, it has been reported that activation of AMP-activated protein kinase (AMPK) by metformin is involved in neuroprotective effects of metformin on central nervous system diseases [20, 26]. Whereas, whether metformin confers neuroprotective effects on HI brain injury via activation of AMPK is obscure. Thus, more investigations should be conducted in the future. Furthermore, as therapeutic hypothermia is already a routine treatment for neonatal encephalopathy and the neuroprotective effects of metformin in the neonatal HI brain injury has been clearly illustrated, it is significant to investigate whether metformin augments hypothermic neuroprotection. Therefore, what are warranted in the following study is to determine neuroprotective effects of metformin-augmented hypothermia compared with hypothermia alone after HI insult.

In summary, our research demonstrated that metformin treatment significantly attenuated brain damage in a neonatal HI brain injury paradigm. The protective effects are likely mediated by inhibition of neuroinflammation and down-regulation of neuronal apoptosis as well as prevention of BBB breakdown by metformin. On the basis of these data in our research, we conclude that a further preclinical investigation of metformin for the therapy or prevention of the neonatal brain damage is warranted.

## MATERIALS AND METHODS

### Neonatal HI injury model and metformin administration

All animal experiments were conducted in compliance with the Guidelines for the Care and Use of Laboratory Animals from the National Institutes of Health and were approved by the Laboratory Animal Ethics Committee of Wenzhou Medical University. We made every efforts to alleviate animal suffering and to minimize the number of animals used. Sprague Dawley (SD) rats were obtained from the Animal Center of the Chinese Academy of Science (Shanghai, China). Adults rats were crossed to deliver litters for subsequent studies, and all animals were housed in an environment of constant temperature under a 12 h light/dark cycle and allowed free access to food and water *ad libitum*. Neonatal hypoxia-ischemia injury model was produced on male rat pups at postnatal day 7 (P7) as previously described [56]. Briefly, rat pups were received a left common carotid artery ligation after anesthesia with 3% isoflurane and the balance of room air. After surgery, pups were recovered for 2 h in their dam and then placed in a hypoxia chamber perfused with a humidified gas mixture of 8% oxygen and 92% nitrogen at a flow rate of 3 L/min. The chamber was partially submerged in a 37.5°C water bath to maintain a constant thermal environment. Pups were returned to their dam after kept in the hypoxia chamber for 2.5 h. For control measurements, a sham group that had ligature placed in an identical fashion without actual artery ligation and without exposure to the hypoxia conditions. Survival rates of animals were 95%. Metformin (Sigma-Aldrich, St. Louis, MO, USA) was dissolved in sterile 0.9% normal saline solution at a concentration of 20 mg/ml at 4°C for future use. A dose of 20 mg/kg metformin was injected subcutaneously into the pups (groups of HI plus metformin) immediately after HI injury [34]. Then, the equivalent dose of metformin was administered at 24 h intervals until the animal euthanized at P14 to assess the long-term neuroprotective effects against neonatal HI injury. The vehicle-treated HI group received the equivalent dose of sterile 0.9% normal saline solution at the same time as the metformin-treated HI groups.

### Brain water content

Rats were sacrificed under deep anesthesia at 24 h after HI injury and the brains in each group were rapidly removed. The hemispheres were separated into ipsilateral and contralateral and immediately weighted on a high precision balance (Denver Instrument, sensitivity ±0.001 g). The hemispheres were then placed in an oven for 72 h at 100°C as previously described [49] and reweighed. Brain edema was determined using the wet/dry method: Percent brain water = [(Wet weight- Dry weight)/ Wet weight]*100%

### Infarct volume measurement

Infarct volume was measured using 2,3,5- Triphenyltetrazolium chloride (TTC) staining as previously described [57]. Briefly, at 24 h after HI injury, rats were perfused via cardiac puncture with 0.9% normal saline under deep anesthesia. The brains were rapidly removed after decapitation, maintained at -80°C for 6 min and then sectioned at 2 mm intervals into 4 coronal slices. After immersion in 1% TTC solution (Sigma-Aldrich, St. Louis, MO) for 15-20 min at 37°C, the brain slices were fixed in 4% paraformaldehyde (PFA) overnight. Photographs of the sections were taken with a digital camera, and the Infarct volumes were traced and measured by Image J software. The percentage of infarction (infarct ratio) was calculated by dividing the infarct area by the total area of the ipsilateral hemisphere [58].

### Histology, immunofluorescence and immunohistochemistry

Rats were deeply anesthetized with 3% isoflurane and transcardially perfused with 20ml of 0.9% normal saline, then with 20 ml of 4% paraformaldehyde (PFA) in 10mM phosphate buffered saline (PBS) at 24 h and 7 d after HI injury. Brains were post-fixed in 4% PFA overnight at 4°C, dehydrated using graded ethanol and xylene, embedded in paraffin and sectioned coronally into 5μm slices for subsequent staining. Tissue sections for histopathological assessment were subjected to hematoxylin and eosin (H&E) staining and Nissl staining following the manufacturer’s instructions. Bright-field images were acquired using light microscopy.

Additional coronal 5μm sections were chosen for immunohistochemistry and immunofluorescence. After deparaffination, rehydration and antigen retrieval by boiling in citrate buffer for 10 minutes, sections were incubated in 3% H_2_O_2_ (30% H_2_O_2_ diluted 1:99 in 80% methanol) for 15 min to block the endogenous peroxidase activity, followed by blocking with 5% bovine serum albumin (BSA) in 10 mM PBS for 30 min at 37°C. The primary antibodies were diluted in 10 mM PBS containing 1% BSA, 0.3% Triton X-100. For immunohistochemistry, the sections obtained at 7 d after HI injury were incubated at 4°C overnight with primary antibodies targeting the following proteins: myelin basic protein (MBP, 1:200, sc-13914, Santa Cruz Biotechnology) and microtubule-associated protein 2 (MAP-2, 1:200, sc-20172, Santa Cruz Biotechnology). Sections were then rinsed three times with PBS and treated with horseradish peroxidase (HRP) conjugated donkey anti-goat secondary antibody (1:1000, sc-2020, Santa Cruz Biotechnology) for 1 h at 37°C. Then, the reaction was stopped with 3, 3-diaminobenzidine (DAB). For immunofluorescence, the sections obtained at 24 h after HI injury were incubated with primary antibodies targeting the following proteins: IL-6 (1:200, sc-1266, Santa Cruz Biotechnology), TNF-α (5 μg/ml, ab9755, Abcam), Iba-1 (1:400, ab5076, Abcam), GFAP (1:200, sc-58766, Santa Cruz Biotechnology), Desmin (1:100, sc-34200, Santa Cruz Biotechnology), PDGFR-β (1:500, ab32570, Abcam). Sections were then rinsed three times with PBS and treated with the AlexaFluor 488 donkey anti-rabbit/goat secondary antibodies (1:1000, Abcam) for 1 h at 37°C. Next, the sections were rinsed three times with PBST, counterstained with 4, 6-diamidino-2-phenylindole (DAPI) for 7 min and finally rinsed twice in PBS and sealed with a coverslip. All the images were captured using a Nikon ECLIPSE Ti microscope (Nikon, Tokyo, Japan). The extent of tissue damage was measured by calculating the amount of surviving tissue in each section. Briefly, brain damage was analyzed using the Image J software (http://imagej.nih.gov/ij/) by outlining both hemispheres on full section images. The ipsilateral MBP area was calculated as percentage for each animal using the following equation: [1- (area ipsliateral MBP staining/area contralateral MBP staining)]×100. The MAP-2 area was determined as same as the calculation method of the MBP area loss.

### TUNEL assay

DNA fragmentation was detected using an In Situ Cell Death Detection Kit (Roche, South San Francisco, CA, USA). According to the standard protocol, sections obtained at 24 h after HI injury were deparaffinised and rehydrated. Then, these sections were treated in a 20 μg/ml proteinase K working solution for 10 min at 37°C. The sections were rinsed three times in PBS, followed by incubation with TUNEL reaction mixture in a dark humidified chamber for 1 h at 37°C. After rinsed three times with PBS, the sections were treated with DAPI for 7 min at room temperature. Negative controls were obtained by omitting the TdT enzyme. Apoptotic cells were characterized by green fluorescence of the nucleus and nuclear membrane according to the manufacturer’s protocol. Quantitation was performed by counting the number of positive cells in five randomly chosen fields within each slide at 400× magnification. The index of apoptosis was calculated as the ratio of the overall number of apoptotic cells to the total number of cells. The results were imaged at 400× using a Nikon ECLIPSE Ti microscope (Nikon, Tokyo, Japan) and analyzed by using Image J software.

### Quantitative real-time RT-PCR

Animals were decapitated under deep anesthesia, the brains were quickly dissected out and separated into the cortex and hippocampus on a bed of ice, and then frozen in liquid nitrogen and stored at -80°C. Total RNA was extracted from the tissue samples using the TriPure Isolation Reagent (Roche, South San Francisco, CA, USA) according to the manufacturer’s instructions. The concentration of total RNA was measured by Nanodrop spectrometry (Thermo Fisher Scientific); only those samples with the OD 260/280 ratio of greater than 1.8 were used. Up to 0.5 μg of RNA was used to synthesize the first strand of cDNA using PrimeScript ™RT Reagent Kit (TaKaRa, Kusatsu, Shiga, Japan) and Bio-Rad MyCycler TM™ Thermal Cycler for the reverse transcription polymerase chain reaction. Reverse transcription products were amplified with the 7900HT Fast Real-Time PCR system in a 10 μl final reaction volume using SYBR Green PCR Master Mix (Bio-Rad, Hercules, CA, USA) under the following conditions: 2 min at 95°C and 30 s at 60°C, followed by a total of 40 cycles of 2 temperature cycles (15 s at 95°C and 30 s at 60°C). Expression level of β-actin gene was used for standardization. The forward and reverse primer sequences are shown in Table 1. The fluorescence threshold value (CT value) was calculated using the SDS Enterprise Database software. The CT values of the genes of interest were first normalized with β-actin of the same sample, and then the gene expression level in vehicle- and metformin-treated groups were calculated and expressed as fold change versus sham group (setting sham as 1).

**Table 1:** Primers used in the studies

Gene	Forward primers	Reverse primers
TNF-α	TACTCCCAGGTTCTCTTCAAGG	GGAGGCTGACTTTCTCCTGGTA
IL-18	AAACCCGCCTGTGTTCGA	TCAGTCTGGTCTGGGATTCGT
IL-1β	CACCTCTCAAGCAGAGCACAG	GGGTTCCATGGTGAAGTCAAC
IL-6	GAGTTGTGCAATGGCAATTC	ACTCCAGAAGACCAGAGCAG
iNOS	AGGCCACCTCGGATATCTCT	GCTTGTCTCTGGGTCCTCTG
COX-2	CGGAGGAGAAGTGGGGTTTAGGAT	TGGGAGGCACTTGCGTTGATGG
β-actin	AAGTCCCTCACCCTCCCAAAAG	AAGCAATGCTGTCACCTTCCC

### Western blot

Rats were deeply anesthetized and then killed by decapitation at 24 h and 7 d after HI injury, cortex and hippocampus were dissected and stored at -80°C immediately until analysis. The samples of cortex and hippocampus were respectively homogenized in a modified RIPA buffer (25 mM Tris-HCl, 150 mM NaCl, 1% Nonidet P-40, 1% sodium deoxycholate, and 0.1% sodium dodecyl sulfate) containing protease inhibitor (GE Healthcare Biosciences, Piscataway, NJ, USA) and further centrifuged at 12000 rpm and 4°C for 10 min to collect the supernatant for protein assay. The extracts above were first quantified with BCA reagents (Beyotime). Equal amount of protein (80 μg) were loaded and separated on a 12% SDS-PAGE gel and transferred onto PVDF membrane (Bio-Rad, Hercules, CA, USA). After blocking with 5% non-fat dry skim milk for 2 h at room temperature, the membranes were incubated with the primary antibody overnight on a shaker at 4°C. The primary antibodies used were P120 (1:1000, ab92514, Abcam), β-Catenin (1:1000, 8480S, Cell Signaling Technology), VE-Cadherin (1:1000, ab33168, Abcam), Occludin (1:1000, 13409, Proteintech), Claudin-5 (1:300, sc-374221, Santa Cruz Biotechnology), PDGFR-β (1:1000, 3169S, Cell Signaling Technology), Desmin (1:1000, 5332S, Cell Signaling Technology), Bax (1:300, sc-493, Santa Cruz Biotechnology), Bcl-2 (1:1000, #2876, Cell Signaling Technology), cleaved caspase 3 (1:1000, 9664S, Cell Signaling Technology), TLR4 (1:1000, ab30667, Abcam), NF-κB (P65, 1:300, sc-7151, Santa Cruz Biotechnology), IκB-α (1:300, sc-371, Santa Cruz Biotechnology), TNF-α (1:3000, ab9755, Abcam), GFAP (1:300, sc-58766, Santa Cruz Biotechnology), MAP-2 (1:1000, #4542S, Cell Signaling Technology), MBP (1:1000, ab40390, Abcam). The membranes were then washed three times in TBST and incubated with appropriate HRP-conjugated secondary antibodies (1:10000, Bioworld) for 1 h at room temperature. Western blotting of β-actin (1:3000, Bioworld, AP0060) was used as an internal control to demonstrate equal protein loading. The signals were visualized by ChemiDocXRS^+^ Imaging System (Bio-Rad). All experiments was repeated at least in triplicate, and the densitometric values of the bands on western blots were quantified by the Image Lab software (Bio-Rad).

### Statistical analysis

Results were presented as the mean ± SEM from three independent experiments. Statistical significance was assessed by using Student’s t test if comparing only two experimental groups or one-way analysis of variance (ANOVA) followed by Turkey *post hoc* test if analysing more than two groups. All statistical analyses were performed using GraphPad Prism 5.0, and *P* value < 0.05 was considered as statistically significant.

## References

[1] Kurinczuk JJ, White-Koning M, Badawi N (2010). Epidemiology of neonatal encephalopathy and hypoxic-ischaemic encephalopathy. Early Hum Dev.

[2] Lai MC, Yang SN (2011). Perinatal hypoxic-ischemic encephalopathy. J Biomed Biotechnol.

[3] Douglas-Escobar M, Weiss MD (2015). Hypoxic-ischemic encephalopathy: a review for the clinician. JAMA Pediatr.

[4] Chiang MC, Jong YJ, Lin CH (2017). Therapeutic hypothermia for neonates with hypoxic ischemic encephalopathy. Pediatr Neonatol.

[5] Shankaran S, Pappas A, McDonald SA, Vohr BR, Hintz SR, Yolton K, Gustafson KE, Leach TM, Green C, Bara R, Petrie Huitema CM, Ehrenkranz RA, Tyson JE (2012). Childhood outcomes after hypothermia for neonatal encephalopathy. N Engl J Med.

[6] Hagberg H, Mallard C, Ferriero DM, Vannucci SJ, Levison SW, Vexler ZS, Gressens P (2015). The role of inflammation in perinatal brain injury. Nat Rev Neurol.

[7] Hedtjarn M, Mallard C, Hagberg H (2004). Inflammatory gene profiling in the developing mouse brain after hypoxia-ischemia. J Cereb Blood Flow Metab.

[8] Lehnardt S, Massillon L, Follett P, Jensen FE, Ratan R, Rosenberg PA, Volpe JJ, Vartanian T (2003). Activation of innate immunity in the CNS triggers neurodegeneration through a Toll-like receptor 4-dependent pathway. Proc Natl Acad Sci U S A.

[9] Zhang P, Cheng G, Chen L, Zhou W, Sun J (2015). Cerebral hypoxia-ischemia increases toll-like receptor 2 and 4 expression in the hippocampus of neonatal rats. Brain Dev.

[10] Yao L, Kan EM, Lu J, Hao A, Dheen ST, Kaur C, Ling EA (2013). Toll-like receptor 4 mediates microglial activation and production of inflammatory mediators in neonatal rat brain following hypoxia: role of TLR4 in hypoxic microglia. J Neuroinflammation.

[11] Obermeier B, Daneman R, Ransohoff RM (2013). Development, maintenance and disruption of the blood-brain barrier. Nat Med.

[12] Madden JA (2012). Role of the vascular endothelium and plaque in acute ischemic stroke. Neurology.

[13] Tu YF, Tsai YS, Wang LW, Wu HC, Huang CC, Ho CJ (2011). Overweight worsens apoptosis, neuroinflammation and blood-brain barrier damage after hypoxic ischemia in neonatal brain through JNK hyperactivation. J Neuroinflammation.

[14] Northington FJ, Chavez-Valdez R, Martin LJ (2011). Neuronal cell death in neonatal hypoxia-ischemia. Ann Neurol.

[15] Rocha-Ferreira E, Hristova M (2016). Plasticity in the neonatal brain following hypoxic-ischaemic injury. Neural Plast.

[16] Ferriero DM (2004). Neonatal brain injury. N Engl J Med.

[17] Northington FJ, Graham EM, Martin LJ (2005). Apoptosis in perinatal hypoxic-ischemic brain injury: how important is it and should it be inhibited?. Brain Res Brain Res Rev.

[18] Martin-Montalvo A, Mercken EM, Mitchell SJ, Palacios HH, Mote PL, Scheibye-Knudsen M, Gomes AP, Ward TM, Minor RK, Blouin MJ, Schwab M, Pollak M, Zhang Y (2013). Metformin improves healthspan and lifespan in mice. Nat Commun.

[19] Eikawa S, Nishida M, Mizukami S, Yamazaki C, Nakayama E, Udono H (2015). Immune-mediated antitumor effect by type 2 diabetes drug, metformin. Proc Natl Acad Sci U S A.

[20] Ashabi G, Khalaj L, Khodagholi F, Goudarzvand M, Sarkaki A (2015). Pre-treatment with metformin activates Nrf2 antioxidant pathways and inhibits inflammatory responses through induction of AMPK after transient global cerebral ischemia. Metab Brain Dis.

[21] Wang C, Liu C, Gao K, Zhao H, Zhou Z, Shen Z, Guo Y, Li Z, Yao T, Mei X (2016). Metformin preconditioning provide neuroprotection through enhancement of autophagy and suppression of inflammation and apoptosis after spinal cord injury. Biochem Biophys Res Commun.

[22] Labuzek K, Liber S, Gabryel B, Adamczyk J, Okopien B (2010). Metformin increases phagocytosis and acidifies lysosomal/endosomal compartments in AMPK-dependent manner in rat primary microglia. Naunyn Schmiedebergs Arch Pharmacol.

[23] Liu Y, Tang G, Li Y, Wang Y, Chen X, Gu X, Zhang Z, Wang Y, Yang GY (2014). Metformin attenuates blood-brain barrier disruption in mice following middle cerebral artery occlusion. J Neuroinflammation.

[24] Ge XH, Zhu GJ, Geng DQ, Zhang HZ, He JM, Guo AZ, Ma LL, Yu DH (2017). Metformin protects the brain against ischemia/reperfusion injury through PI3K/Akt1/JNK3 signaling pathways in rats. Physiol Behav.

[25] Zhang D, Xuan J, Zheng BB, Zhou YL, Lin Y, Wu YS, Zhou YF, Huang YX, Wang Q, Shen LY, Mao C, Wu Y, Wang XY (2017). Metformin improves functional recovery after spinal cord injury via autophagy flux stimulation. Mol Neurobiol.

[26] Takata F, Dohgu S, Matsumoto J, Machida T, Kaneshima S, Matsuo M, Sakaguchi S, Takeshige Y, Yamauchi A, Kataoka Y (2013). Metformin induces up-regulation of blood-brain barrier functions by activating AMP-activated protein kinase in rat brain microvascular endothelial cells. Biochem Biophys Res Commun.

[27] Jin Q, Cheng J, Liu Y, Wu J, Wang X, Wei S, Zhou X, Qin Z, Jia J, Zhen X (2014). Improvement of functional recovery by chronic metformin treatment is associated with enhanced alternative activation of microglia/macrophages and increased angiogenesis and neurogenesis following experimental stroke. Brain Behav Immun.

[28] Qi B, Hu L, Zhu L, Shang L, Sheng L, Wang X, Liu N, Wen N, Yu X, Wang Q, Yang Y (2016). Metformin attenuates cognitive impairments in hypoxia-ischemia neonatal rats via improving remyelination. Cell Mol Neurobiol.

[29] Dalkara T, Gursoy-Ozdemir Y, Yemisci M (2011). Brain microvascular pericytes in health and disease. Acta Neuropathol.

[30] Sweeney MD, Ayyadurai S, Zlokovic BV (2016). Pericytes of the neurovascular unit: key functions and signaling pathways. Nat Neurosci.

[31] Shen H, Hu X, Liu C, Wang S, Zhang W, Gao H, Stetler RA, Gao Y, Chen J (2010). Ethyl pyruvate protects against hypoxic-ischemic brain injury via anti-cell death and anti-inflammatory mechanisms. Neurobiol Dis.

[32] Azzopardi DV, Strohm B, Edwards AD, Dyet L, Halliday HL, Juszczak E, Kapellou O, Levene M, Marlow N, Porter E, Thoresen M, Whitelaw A, Brocklehurst P (2009). Moderate hypothermia to treat perinatal asphyxial encephalopathy. N Engl J Med.

[33] Fernandez-Lopez D, Natarajan N, Ashwal S, Vexler ZS (2014). Mechanisms of perinatal arterial ischemic stroke. J Cereb Blood Flow Metab.

[34] Dadwal P, Mahmud N, Sinai L, Azimi A, Fatt M, Wondisford FE, Miller FD, Morshead CM (2015). Activating endogenous neural precursor cells using metformin leads to neural repair and functional recovery in a model of childhood brain injury. Stem Cell Reports.

[35] Liu F, McCullough LD (2013). Inflammatory responses in hypoxic ischemic encephalopathy. Acta Pharmacol Sin.

[36] Girard S, Kadhim H, Roy M, Lavoie K, Brochu ME, Larouche A, Sebire G (2009). Role of perinatal inflammation in cerebral palsy. Pediatr Neurol.

[37] Teo JD, Morris MJ, Jones NM (2015). Hypoxic postconditioning reduces microglial activation, astrocyte and caspase activity, and inflammatory markers after hypoxia-ischemia in the neonatal rat brain. Pediatr Res.

[38] Chen CY, Sun WZ, Kang KH, Chou HC, Tsao PN, Hsieh WS, Fu WM (2015). Hypoxic preconditioning suppresses glial activation and neuroinflammation in neonatal brain insults. Mediators Inflamm.

[39] Liu DL, Zhao LX, Zhang S, Du JR (2016). Peroxiredoxin 1-mediated activation of TLR4/NF-kappaB pathway contributes to neuroinflammatory injury in intracerebral hemorrhage. Int Immunopharmacol.

[40] Liu J, Chen Q, Jian Z, Xiong X, Shao L, Jin T, Zhu X, Wang L (2016). Daphnetin protects against cerebral ischemia/reperfusion injury in mice via inhibition of TLR4/NF-κB signaling pathway. Biomed Res Int.

[41] Tang SC, Arumugam TV, Xu X, Cheng A, Mughal MR, Jo DG, Lathia JD, Siler DA, Chigurupati S, Ouyang X, Magnus T, Camandola S, Mattson MP (2007). Pivotal role for neuronal Toll-like receptors in ischemic brain injury and functional deficits. Proc Natl Acad Sci U S A.

[42] Cao CX, Yang QW, Lv FL, Cui J, Fu HB, Wang JZ (2007). Reduced cerebral ischemia-reperfusion injury in Toll-like receptor 4 deficient mice. Biochem Biophys Res Commun.

[43] Nijboer CH, Heijnen CJ, Groenendaal F, van Bel F, Kavelaars A (2009). Alternate pathways preserve tumor necrosis factor-alpha production after nuclear factor-kappaB inhibition in neonatal cerebral hypoxia-ischemia. Stroke.

[44] van der Kooij MA, Nijboer CH, Ohl F, Groenendaal F, Heijnen CJ, van Bel F, Kavelaars A (2010). NF-kappa B inhibition after neonatal cerebral hypoxia-ischemia improves long-term motor and cognitive outcome in rats. Neurobiol Dis.

[45] Brown J, Wang H, Hajishengallis GN, Martin M (2011). TLR-signaling networks: an integration of adaptor molecules, kinases, and cross-talk. J Dent Res.

[46] Daneman R (2012). The blood-brain barrier in health and disease. Ann Neurol.

[47] Yang Y, Rosenberg GA (2011). Blood-brain barrier breakdown in acute and chronic cerebrovascular disease. Stroke.

[48] Ek CJ, D’Angelo B, Baburamani AA, Lehner C, Leverin AL, Smith PL, Nilsson H, Svedin P, Hagberg H, Mallard C (2015). Brain barrier properties and cerebral blood flow in neonatal mice exposed to cerebral hypoxia-ischemia. J Cereb Blood Flow Metab.

[49] Zhang W, Zhang H, Mu H, Zhu W, Jiang X, Hu X, Shi Y, Leak RK, Dong Q, Chen J, Gao Y (2016). Omega-3 polyunsaturated fatty acids mitigate blood-brain barrier disruption after hypoxic-ischemic brain injury. Neurobiol Dis.

[50] Li L, McBride DW, Doycheva D, Dixon BJ, Krafft PR, Zhang JH, Tang J (2015). G-CSF attenuates neuroinflammation and stabilizes the blood-brain barrier via the PI3K/Akt/GSK-3β signaling pathway following neonatal hypoxia-ischemia in rats. Exp Neurol.

[51] Daneman R, Zhou L, Kebede AA, Barres BA (2010). Pericytes are required for blood-brain barrier integrity during embryogenesis. Nature.

[52] Armulik A, Genove G, Mae M, Nisancioglu MH, Wallgard E, Niaudet C, He L, Norlin J, Lindblom P, Strittmatter K, Johansson BR, Betsholtz C (2010). Pericytes regulate the blood-brain barrier. Nature.

[53] Zhu C, Wang X, Xu F, Bahr BA, Shibata M, Uchiyama Y, Hagberg H, Blomgren K (2005). The influence of age on apoptotic and other mechanisms of cell death after cerebral hypoxia-ischemia. Cell Death Differ.

[54] Siddiqui WA, Ahad A, Ahsan H (2015). The mystery of BCL2 family: Bcl-2 proteins and apoptosis: an update. Arch Toxicol.

[55] Thornton C, Rousset CI, Kichev A, Miyakuni Y, Vontell R, Baburamani AA, Fleiss B, Gressens P, Hagberg H (2012). Molecular mechanisms of neonatal brain injury. Neurol Res Int.

[56] Vannucci RC, Connor JR, Mauger DT, Palmer C, Smith MB, Towfighi J, Vannucci SJ (1999). Rat model of perinatal hypoxic-ischemic brain damage. J Neurosci Res.

[57] Tian SF, Yang HH, Xiao DP, Huang YJ, He GY, Ma HR, Xia F, Shi XC (2013). Mechanisms of neuroprotection from hypoxia-ischemia (HI) brain injury by up-regulation of cytoglobin (CYGB) in a neonatal rat model. J Biol Chem.

[58] Yanamoto H, Hong SC, Soleau S, Kassell NF, Lee KS (1996). Mild postischemic hypothermia limits cerebral injury following transient focal ischemia in rat neocortex. Brain Res.

